# Supplementary material for: Structural Features and Phylogenetic Implications of 11 New Mitogenomes of Typhlocybinae (Hemiptera: Cicadellidae)
Source: Insects. 2021 Jul 28;12(8):678. doi: 10.3390/insects12080678 (PMC8396557; doi:10.3390/insects12080678)
Supplement: Supplementary file 1 [file insects-12-00678-s001.zip › insects-1286606-supplementary.pdf]

Table S1. Collection information of adult specimens in this study.

Name	Locality	Date	Collector
Shaddai sp.	Ziwuling mountains, Shaanxi Province, China	25 JUN 2019	Meixia Yang
Sobrala sp.	Baishi Town, Chongqing City, China	20 July 2019	Shuanghu Lin
Dikraneura (*D.*) *zlata*	Qingping Town, Zhangjiajie City, Hunan Province, China	10 Aug 2019	Shuanghu Lin
Dikraneurini sp.	Baishi Town, Chongqing City, China	20 July 2019	Shuanghu Lin
Alebroides salicis	Qingping Town, Zhangjiajie City, Hunan Province, China	10 Aug 2019	Shuanghu Lin
Empoasca serrata	Ziwuling mountains, Shaanxi Province, China	25 JUN 2019	Meixia Yang
Elbelus tripunctatus	Leigong Mountain, Kaili City, Guizhou Province, China	6 Aug 2019	Shuanghu Lin
Kaukania anser	Fanjing mount, Tongren City, Guizhou Province, China	4 Aug 2019	Shuanghu Lin
Eupteryx (*E.*) *adspersa*	Ziwuling mountains, Shaanxi Province, China	25 JUN 2019	Meixia Yang
Eurhadina jarrary	Tongwan Town, Huaihua City, Hunan Province, China	8 Aug 2019	Shuanghu Lin
Yangisunda tiani	Baiguo Town, Enshi City, Hubei Province	17 July 2019	Shuanghu Lin

Table S2. The best partitioning schemes and models for Maximum likelihood (ML) method based on four datasets selected by PartitionFinder.

Dataset	Partitioning scheme	Models
PCG123	P1: (*cytb*_pos1, *cox1*_pos1, *atp6*_pos1, *cox3*_pos1, *cox2*_pos1)	GTR+I+G
	P2: (*nad2*_pos2, *atp8*_pos2, *nad3*_pos2, *nad6*_pos2, *cox2*_pos2, *atp6*_pos2）	TVM+I+G
	P3: (*atp6*_pos3, *atp8*_pos3, *nad3*_pos3, *nad6*_pos3, *cytb*_pos3, *cox2*_pos3, *cox1*_pos3, *cox3*_pos3）	HKY+I+G
	P4: (*nad2*_pos1, *atp8*_pos1, *nad3*_pos1, *nad6*_pos1）	TIM+I+G
	P5: (*cox1*_pos2, *cox3*_pos2, *cytb*_pos2）	TVM+I+G
	P6: (*nad1*_pos1, *nad4L*_pos1, *nad5*_pos1, *nad4*_pos1）	GTR+I+G
	P7: (*nad1*_pos2, *nad4L*_pos2, *nad5*_pos2, *nad4*_pos2）	TVM+I+G
	P8: (*nad1*_pos3）	HKY+G
	P9: (*nad2*_pos3）	HKY+G
	P10: (*nad4L*_pos3, *nad5*_pos3, *nad4*_pos3)	HKY+G
PCG123R	P1: (*cytb*_pos1, *cox1*_pos1, *atp6*_pos1, *cox2*_pos1, *cox3*_pos1)	GTR+I+G
	P2: (*nad2*_pos2, *atp8*_pos2, *nad3*_pos2, *nad6*_pos2, *atp6*_pos2, *cox2*_pos2)	TVM+I+G
	P3: (*atp6*_pos3, *atp8*_pos3, *nad6*_pos3, *nad3*_pos3, *cytb*_pos3, *cox2*_pos3, *cox3*_pos3, *cox1*_pos3)	HKY+I+G
	P4: (*nad2*_pos1, *atp8*_pos1, *nad3*_pos1, *nad6*_pos1)	TIM+I+G
	P5: (*cox1*_pos2, *cytb*_pos2, *cox3*_pos2)	TVM+I+G
	P6: (*nad1*_pos1, *nad4L*_pos1, *nad5*_pos1, *nad4*_pos1)	GTR+I+G
	P7: (*nad1*_pos2, *nad4L*_pos2, *nad4*_pos2, *nad5*_pos2)	TVM+I+G
	P8: (*nad1*_pos3)	HKY+G
	P9: (*nad2*_pos3)	HKY+G
	P10: (*nad4L*_pos3, *nad4*_pos3, *nad5*_pos3)	HKY+G
	P11: (*rrnL, rrnS*)	TVM+I+G
PCG12	P1: (*nad6*, *nad3*, *atp6*)	TVM+I+G
	P2: (*atp8*, *nad2*)	TVM+I+G
	P3: (*cox1*, *cytb*)	GTR+I+G
	P4: (*cox3*, *cox2*)	K81UF+I+G
	P5: (*nad1*)	K81UF+G
	P6: (*nad4L*, *nad4*, *nad5*)	TVM+I+G

Table S3. The best partitioning schemes and models for Bayesian inference (BI) method based on four datasets selected by PartitionFinder.

Dataset	Partitioning scheme	Models
PCG123	P1: (*nad2*_pos1, *atp8*_pos1, *atp6*_pos1, *nad3*_pos1, *nad6*_pos1)	GTR+I+G
	P2: (*atp6*_pos2, *nad3*_pos2, *nad6*_pos2, *atp8*_pos2, *nad2*_pos2)	GTR+I+G
	P3: (*atp6*_pos3, *atp8*_pos3, *nad6*_pos3, *nad3*_pos3, *cytb*_pos3, *cox2*_pos3, *cox3*_pos3, *cox1*_pos3)	HKY+I+G
	P4: (*cox2*_pos1, *cox3*_pos1, *cytb*_pos1, *cox1*_pos1)	GTR+I+G
	P5: (*cox1*_pos2, *cytb*_pos2, *cox2*_pos2, *cox3*_pos2)	GTR+I+G
	P6: (*nad1*_pos1, *nad4L*_pos1, *nad4*_pos1, *nad5*_pos1)	GTR+I+G
	P7: (*nad4*_pos2, *nad5*_pos2, *nad1*_pos2, *nad4L*_pos2)	GTR+I+G
	P8: (*nad1*_pos3)	HKY+G
	P9: (*nad2*_pos3)	HKY+G
	P10: (*nad4L*_pos3, *nad5*_pos3, *nad4*_pos3)	HKY+G
PCG123R	P1: (*nad2*_pos1, *atp8*_pos1, *atp6*_pos1, *nad3*_pos1, *nad6*_pos1)	GTR+I+G
	P2: (*atp6*_pos2, *nad6*_pos2, *nad3*_pos2, *atp8*_pos2, *nad2*_pos2)	GTR+I+G
	P3: (*atp6*_pos3, *atp8*_pos3, *nad3*_pos3, *nad6*_pos3, *cytb*_pos3, *cox2*_pos3, *cox3*_pos3, *cox1*_pos3)	HKY+I+G
	P4: (*cox2*_pos1, *cox3*_pos1, *cytb*_pos1, *cox1*_pos1)	GTR+I+G
	P5: (*cox1*_pos2, *cytb*_pos2, *cox2*_pos2, *cox3*_pos2)	GTR+I+G
	P6: (*nad1*_pos1, *nad4L*_pos1, *nad5*_pos1, *nad4*_pos1)	GTR+I+G
	P7: (*nad1*_pos2, *nad4L*_pos2, *nad4*_pos2, *nad5*_pos2)	GTR+I+G
	P8: (*nad1*_pos3)	HKY+G
	P9: (*nad2*_pos3)	HKY+G
	P10: (*nad4L*_pos3, *nad5*_pos3, *nad4*_pos3)	HKY+G
	P11: (*rrnL*, *rrnS*)	GTR+I+G
PCG12	P1: (*nad3*, *atp6*, *cox2*, *cox3*)	GTR+I+G
	P2: (*nad6*, *atp8*, *nad2*)	GTR+I+G
	P3: (*cox1*, *cytb*)	GTR+I+G
	P4: (*nad1*)	GTR+G
	P5: (*nad4L*, *nad4*, *nad5*)	GTR+I+G

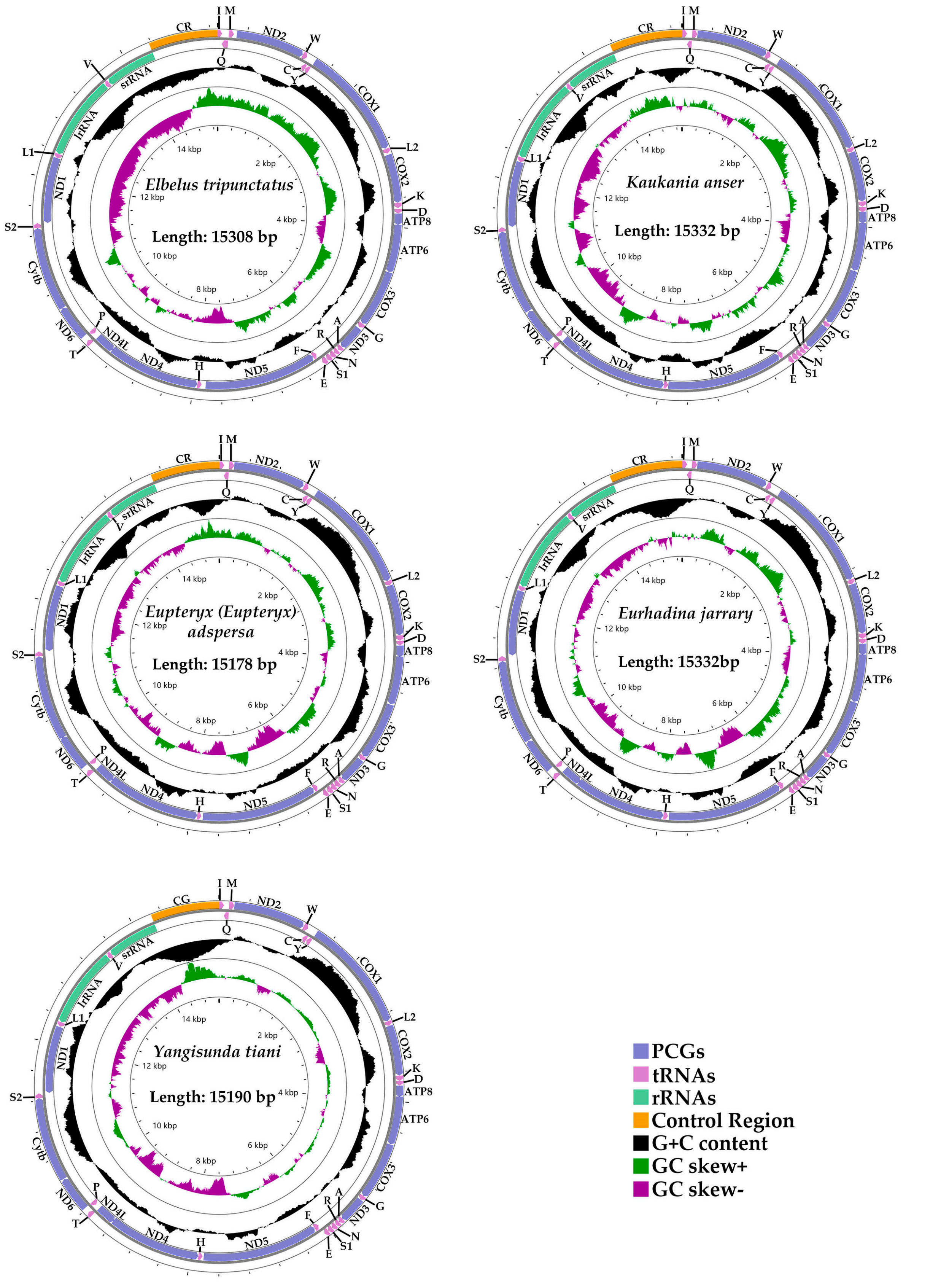

Figure S1. Circular map of the mitochondrial genome of *Elbelus tripunctatus*, *Kaukania anser*, *Eupteryx* (*Eupteryx*) *adspersa*, *Eurhadina jarrary* and *Yangisunda tiani*.

Table S4. Nucleotide composition and skewness of eleven Typhlocybinae spp. mitogenomes. Note: (*Shaddai* sp. (*S1.*); *Sobrala* sp. (*S2.*); *Dikraneura* (*D.*) *zlata* (*D1.*); Dikraneurini sp. (*D2.*); *Alebroides salicis* (*A.*); *Empoasca serrata* (*E1.*); *Elbelus tripunctatus* (*E2.*); *Kaukania anser* (*K.*); *Eupteryx* (*E.*) *adspersa* (*E3.*); *Eurhadina jarrary* (*E4.*); *Yangisunda tiani* (*Y.*)).

Regions	Species	Size (bp)	T%	C%	A%	G%	AT(%)	GC(%)	AT Skew	GC Skew
Full genome	*S1.*	17575	34.8	12.6	41.3	11.3	76.1	23.9	0.085	-0.054
	S2.	16732	34.3	13.9	40.5	11.3	74.8	25.2	0.083	-0.103
	D1.	15330	34.7	13.3	42.0	10.0	76.7	23.3	0.095	-0.142
	D2.	15306	30.2	14.3	45.2	10.3	75.4	24.6	0.199	-0.163
	A.	15890	37.4	13.3	37.7	11.6	75.1	24.9	0.004	-0.068
	E1.	15131	41.0	12.0	35.5	11.4	76.6	23.4	0.072	-0.026
	E2.	15308	36.3	12.0	41.1	10.6	77.4	22.6	0.062	-0.062
	K.	15345	37.4	12.3	39.6	10.6	77.0	23.0	0.029	-0.074
	E3.	15178	34.3	12.2	43.3	10.2	77.6	22.4	0.116	-0.089
	E4.	15332	32.9	12.9	44.6	9.6	77.5	22.5	0.151	-0.147
	Y.	15190	32.1	12.5	46.3	9.1	78.4	21.6	0.181	-0.157
PCGs	*S1.*	10854	43.9	11.4	32.3	12.4	76.2	23.8	-0.152	0.042
	S2.	10956	42.6	12.6	31.8	13.1	74.3	25.7	-0.145	0.019
	D1.	10971	43.4	11.9	32.8	11.9	76.2	23.8	-0.139	0
	D2.	10962	41.5	13.5	32.6	12.4	74.1	25.9	-0.120	-0.042
	A.	10929	44.9	11.8	31.0	12.3	75.9	24.1	-0.183	0.021
	E1.	10947	43.8	12.7	30.4	13.1	74.2	25.8	-0.181	0.016
	E2.	10839	45.0	11.4	31.1	12.6	76.0	24.0	-0.183	0.050
	K.	10959	44.7	12.2	31.0	12.1	75.7	24.3	-0.181	-0.004
	E3.	10935	42.7	11.8	33.3	12.2	76.1	23.9	-0.124	0.017
	E4.	10932	42.0	12.3	33.8	11.9	75.8	24.2	-0.108	-0.017
	Y.	10944	42.2	11.8	34.3	11.7	76.5	23.5	-0.103	-0.004
1st codon position	*S1.*	3618	36.6	10.7	35.0	17.6	71.7	28.3	-0.022	0.244
	S2.	3652	36.1	11.2	35.4	17.3	71.5	28.5	-0.025	0.214
	D1.	3657	36.6	11.0	35.3	17.1	71.9	28.1	-0.018	0.217
	D2.	3654	35.9	11.9	35.5	16.7	71.4	28.6	-0.006	0.168
	A.	3643	37.2	11.2	34.8	16.8	72.0	28.0	-0.033	0.20
	E1.	3649	36.7	11.6	34.5	17.2	71.2	28.8	-0.031	0.194
	E2.	3613	38.0	10.5	34.7	16.7	72.7	27.3	-0.045	0.227
	K.	3653	37.2	11.2	36.5	15.1	73.7	26.3	-0.009	0.148
	E3.	3645	35.6	11.2	35.8	17.3	71.5	28.5	0.003	0.214
	E4.	3644	35.3	11.1	37.1	16.5	72.4	27.6	0.025	0.196
	Y.	3648	35.9	11.1	36.5	16.5	72.4	27.6	0.008	0.196
2nd codon position	*S1.*	3618	47.9	17.2	21.1	13.7	69.0	31.0	-0.388	-0.113
	S2.	3652	48.3	17.6	20.5	13.6	68.8	31.2	-0.404	-0.128
	D1.	3657	48.7	17.3	20.6	13.5	69.2	30.8	-0.406	-0.123
	D2.	3654	47.7	18.0	20.9	13.4	68.6	31.4	-0.391	-0.146
	A.	3643	48.7	17.0	21.1	13.2	69.7	30.3	-0.396	-0.125
	E1.	3649	48.5	17.0	20.9	13.6	69.4	30.6	-0.398	-0.111
	E2.	3613	49.0	17.0	20.4	13.6	69.4	30.6	-0.412	-0.111
	K.	3653	49.1	16.7	21.0	13.2	70.1	29.9	-0.401	-0.117
	E3.	3645	48.3	17.1	20.9	13.7	69.2	30.8	-0.396	-0.110
	E4.	3644	47.7	17.7	20.7	13.9	68.4	31.6	-0.395	-0.120
	Y.	3648	47.6	17.8	20.8	13.7	68.4	31.6	-0.392	-0.130
3rd codon position	*S1.*	3618	47.0	6.2	40.8	6.0	87.8	12.2	-0.071	-0.016
	S2.	3652	43.2	9.1	39.4	8.3	82.6	17.4	-0.046	-0.046
	D1.	3657	44.9	7.5	42.4	5.2	87.3	12.7	-0.029	-0.181
	D2.	3654	40.9	10.6	41.5	7.0	82.4	17.6	0.007	-0.205
	A.	3643	48.9	7.1	37.2	6.9	86.1	13.9	-0.136	-0.014
	E1.	3649	46.1	9.6	35.8	8.5	81.9	18.1	-0.126	-0.061
	E2.	3613	47.9	6.6	38.1	7.4	86.0	14.0	-0.114	0.057
	K.	3653	47.8	8.9	35.4	7.9	83.2	16.8	-0.149	-0.060
	E3.	3645	44.2	6.8	43.2	5.7	87.5	12.5	-0.011	-0.088
	E4.	3644	43.0	8.1	43.6	5.2	86.6	13.4	0.007	-0.216
	Y.	3648	42.9	6.6	45.7	4.9	88.6	11.4	0.032	-0.149

Table S4. Cont.

Regions	Species	Size (bp)	T%	C%	A%	G%	AT(%)	GC(%)	AT Skew	GC Skew
tRNAs	*S1.*	1424	38.8	9.6	38.1	13.6	76.8	23.2	-0.009	0.172
	S2.	1383	38.3	9.5	39.7	12.5	78.0	22.0	0.018	0.136
	D1.	1425	37.7	10.0	39.6	12.7	77.3	22.7	0.025	0.119
	D2.	1420	37.0	10.6	39.1	13.3	76.1	23.9	0.028	0.113
	A.	1422	40.2	9.1	38.7	11.9	79.0	21.0	-0.019	0.133
	E1.	1417	39.7	9.7	38.0	12.6	77.6	22.4	-0.022	0.129
	E2.	1448	39.8	9.3	37.3	13.7	77.1	22.9	-0.032	0.192
	K.	1444	40.3	8.9	38.7	12.1	79.0	21.0	-0.020	0.152
	E3.	1441	38.4	9.2	39.9	12.5	78.3	21.7	0.019	0.152
	E4.	1455	38.7	9.2	39.5	12.6	78.2	21.8	0.010	0.156
	Y.	1430	38.5	9.3	39.4	12.8	77.9	22.1	0.012	0.158
rRNAs	*S1.*	1869	45.8	7.6	35.5	11.1	81.3	18.7	-0.127	0.187
	S2.	1805	46.0	7.4	34.3	12.2	80.3	19.7	-0.146	0.244
	D1.	1860	46.5	6.8	34.6	12.1	81.1	18.9	-0.147	0.280
	D2.	1852	50.9	7.7	28.9	12.6	79.8	20.2	-0.276	0.243
	A.	1855	45.7	7.4	36.3	11.0	81.9	18.1	-0.115	0.199
	E1.	1868	45.2	7.4	36.7	10.7	81.9	18.1	-0.104	0.182
	E2.	1944	51.9	6.6	30.1	11.5	81.9	18.1	-0.266	0.271
	K.	1956	49.1	7.3	32.9	10.7	82.0	18.0	-0.198	0.189
	E3.	1873	47.5	7.0	35.5	10.0	83.0	17.0	-0.145	0.176
	E4.	1920	47.9	6.7	33.8	11.6	81.7	18.3	-0.173	0.268
	Y.	1882	49.5	6.4	32.9	11.2	82.4	17.6	-0.201	0.273
Control region	*S1.*	3328	29.3	13.9	43.0	13.8	72.3	27.7	0.189	-0.004
	S2.	2601	24.6	15.1	36.9	13.5	71.4	28.6	0.172	-0.056
	D1.	1085	36.1	12.9	37.9	13.1	74.0	26.0	0.024	0.008
	D2.	1080	34.4	11.9	45.0	8.8	79.4	20.6	0.134	-0.150
	A.	1697	27.5	23.7	31.9	17.0	59.3	40.7	0.075	-0.165
	E1.	935	68.1	3.6	24.6	3.6	92.7	7.3	-0.469	0
	E2.	933	36.3	8.8	46.5	8.4	82.9	17.1	0.123	-0.023
	K.	982	38.5	9.9	40.9	10.7	79.4	10.7	0.030	0.075
	E3.	923	41.1	8.2	42.3	8.5	83.3	16.7	0.014	0.018
	E4.	989	39.5	7.8	47.2	5.5	86.8	13.2	0.089	-0.174
	Y.	920	33.9	2.6	59.9	3.6	93.8	6.2	0.277	0.161

Table S5. Start and stop codons of the mitochondrial genomes. Note: (*Shaddai* sp. (*S1.*); *Sobrala* sp. (*S2.*); *Dikraneura* (*D.*) *zlata* (*D1.*); Dikraneurini sp. (*D2.*); *Alebroides salicis* (*A.*); *Empoasca serrata* (*E1.*); *Elbelus tripunctatus* (*E2.*); *Kaukania anser* (*K.*); *Eupteryx* (*E.*) *adspersa* (*E3.*); *Eurhadina jarrary* (*E4.*); *Yangisunda tiani* (*Y.*)).

Gene \ Species	*S1.*	*S2.*	*D1.*	*D2.*	*A.*	*E1.*	*E2.*	*K.*	*E3.*	*E4.*	*Y.*
nad2	ATA/TAA	ATA/TAA	ATA/TAA	ATA/TAA	ATT/TAA	ATT/TAA	ATT/TAG	ATA/TAA	ATA/TAG	ATA/TAA	ATA/TAA
cox1	ATG/TAA	ATG/TAA	ATG/T	ATG/TAG	ATG/T	ATG/T	ATG/TAA	ATG/TAA	ATG/TAA	ATG/TAA	ATG/T
cox2	ATA/T	ATT/T	ATT/T	ATA/T	ATG/T	GTG/T	ATT/T	ATG/T	ATT/T	TTG/T	ATT/T
atp8	TTG/TAA	TTG/TAA	TTG/TAA	ATT/TAA	GTG/TAA	ATC/TAA	TTG/TAG	ATT/TAA	TTG/TAA	TTG/TAA	TTG/TAA
atp6	ATA/TAA	ATG/T	ATG/TAA	ATG/TAA	ATG/TAA	ATG/TAA	ATA/TAA	ATT/TAA	ATG/TAA	ATG/TAG	ATG/TAA
cox3	ATG/TAA	ATG/TAA	ATG/TAA	ATG/TAA	ATG/TAA	ATG/TAA	ATG/TAA	ATG/TAA	ATG/T	ATG/T	ATG/T
nad3	ATA/T	ATA/T	ATT/TAG	ATA/TAA	ATT/TAA	ATA/TAA	ATT/TAA	ATA/TAA	ATT/TAA	ATA/TAA	ATA/TAA
nad5	TTG/TAA	ATT/TAA	TTG/T	TTG/T	TTG/T	TTG/T	ATT/TAA	TTG/T	TTG/T	ATT/T	ATT/T
nad4	ATA/TAA	ATG/TAA	ATG/T	ATG/T	ATG/TAA	ATG/TAA	ATG/T	ATG/T	ATG/TAA	ATG/TAA	ATG/TAA
nad4L	ATG/TAG	ATG/TAA	ATA/TAA	ATG/TAA	ATG/TAA	ATG/TAA	ATA/TAA	ATG/TAA	ATG/TAA	ATG/TAG	ATG/TAA
nad6	ATT/TAA	ATT/TAA	ATC/TAA	ATT/TAA	ATT/TAA	ATA/TAA	ATA/TAA	ATT/TAA	ATT/TAA	ATT/TAA	ATT/TAA
cytb	ATG/TAG	ATG/TAG	ATG/TAA	ATG/TAA	ATG/TAG	ATG/TAA	ATG/TAA	ATG/TAG	ATG/TAG	ATG/TAG	ATG/TAG
nad1	ATT/TAA	ATT/T	ATA/TAA	ATA/TAA	ATT/TAA	ATT/TAA	ATA/TAA	ATT/TAA	ATT/TAA	ATT/TAA	ATT/TAA

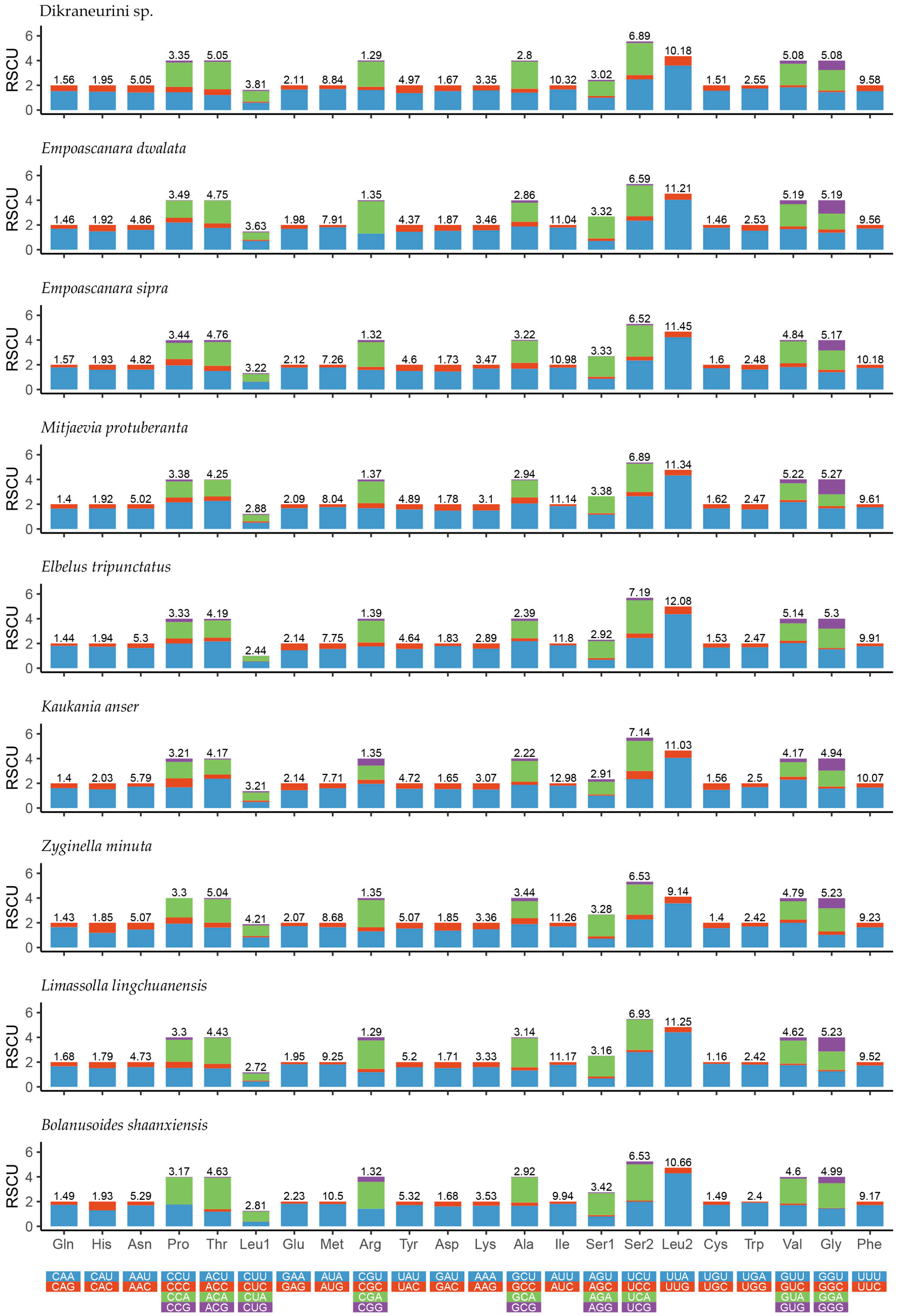

Figure S2. Relative synonymous codon usage (RSCU) in the mitogenomes of Dikraneurini sp., *Empoascanara dwalata*, *Empoascanara sipra*, *Mitjaevia protuberanta*, *Elbelus tripunctatus*, *Kaukania anser*, *Zyginella minuta*, *Limassolla lingchuanensis* and *Bolanusoides shaanxiensis*.

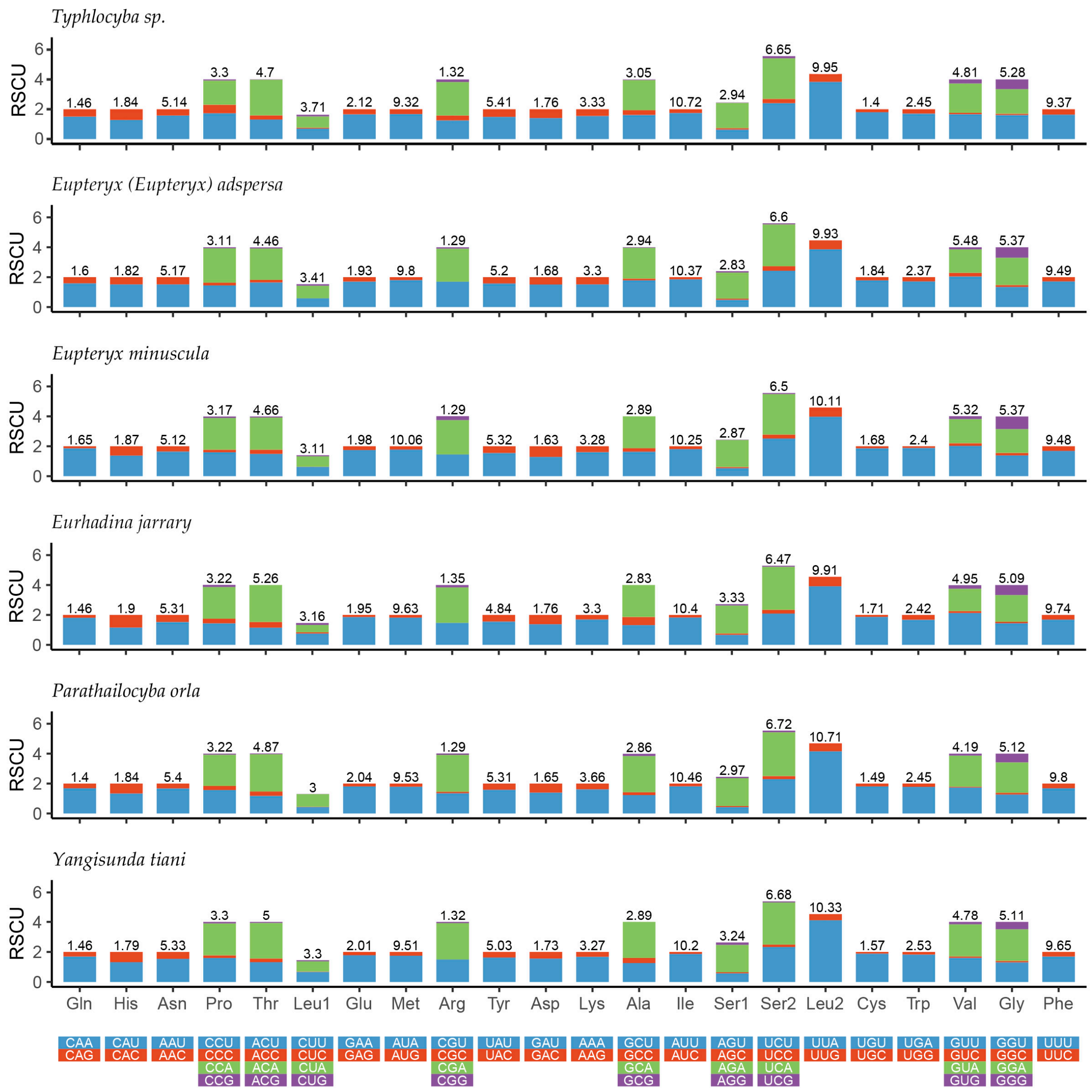

Figure S3. Relative synonymous codon usage (RSCU) in the mitogenomes of *Typhlocyba* sp., *Eupteryx* (*E.*) *adspersa*, *Eupteryx minuscula*, *Eurhadina jarrary*, *Parathailocyba orla* and *Yangisunda tiani*.

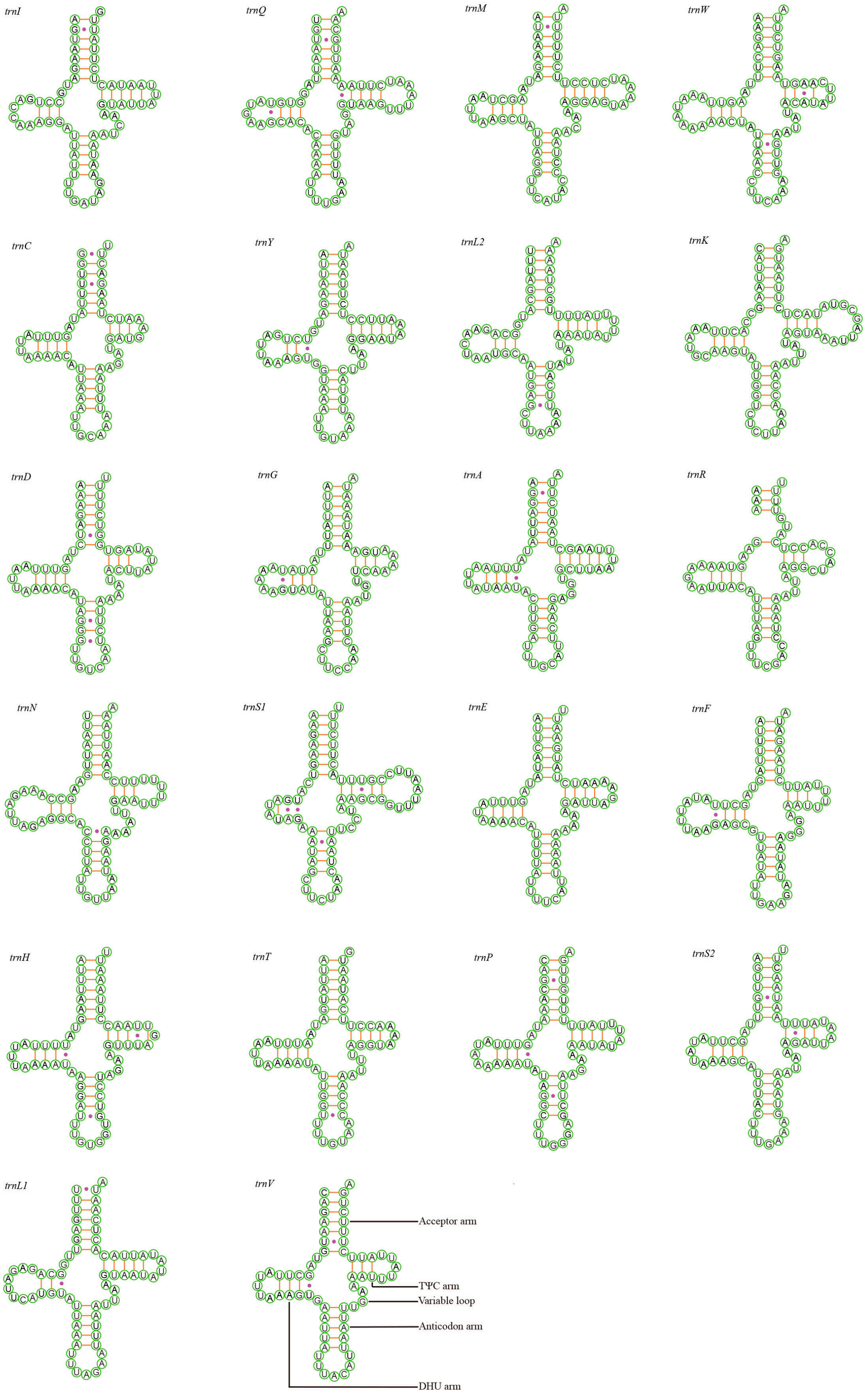

Figure S4. Predicted secondary cloverleaf structure for the tRNAs of *Dikraneura* (*D.*) *zlata*.

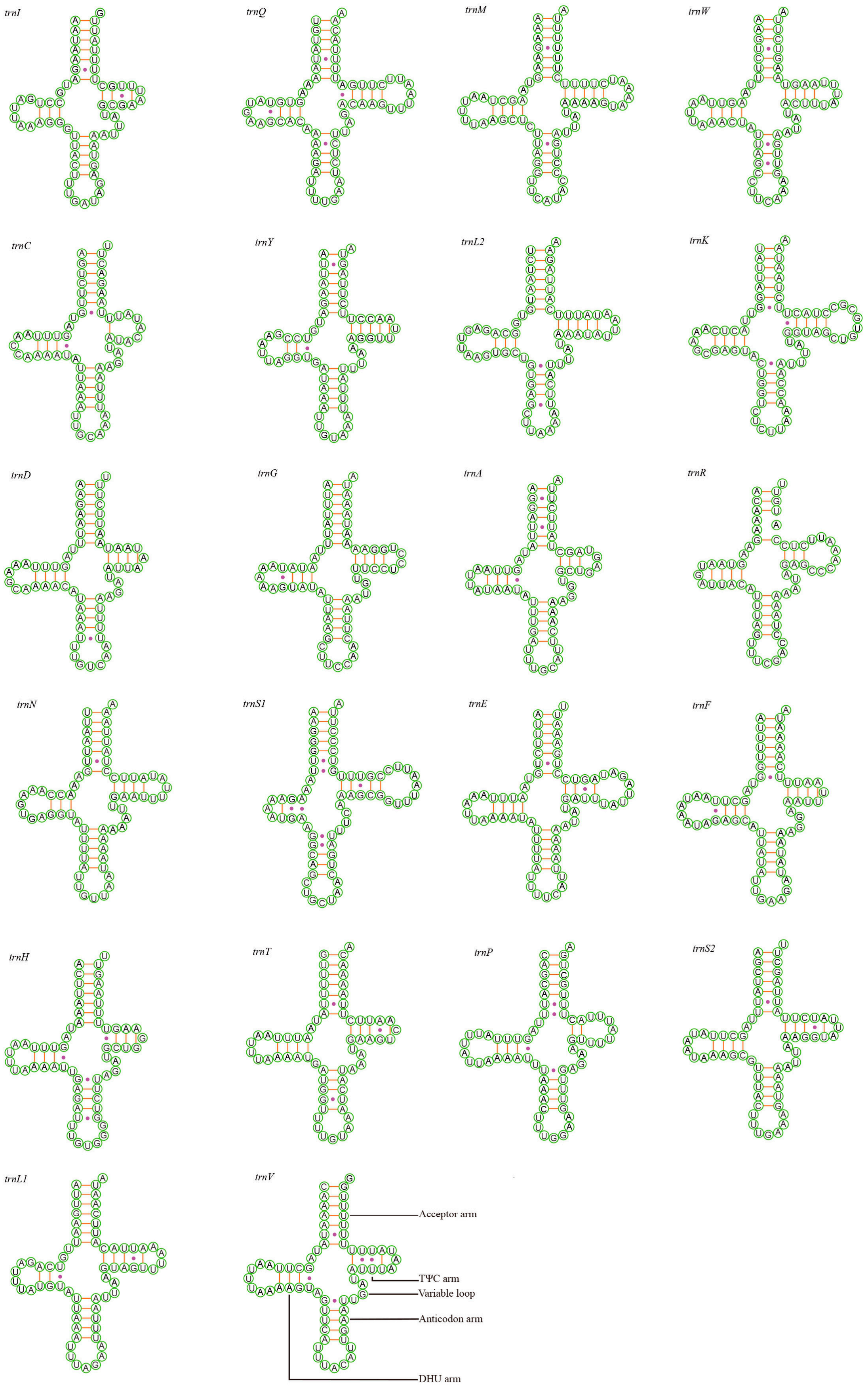

Figure S5. Predicted secondary cloverleaf structure for the tRNAs of *Elbelus tripunctatus*.

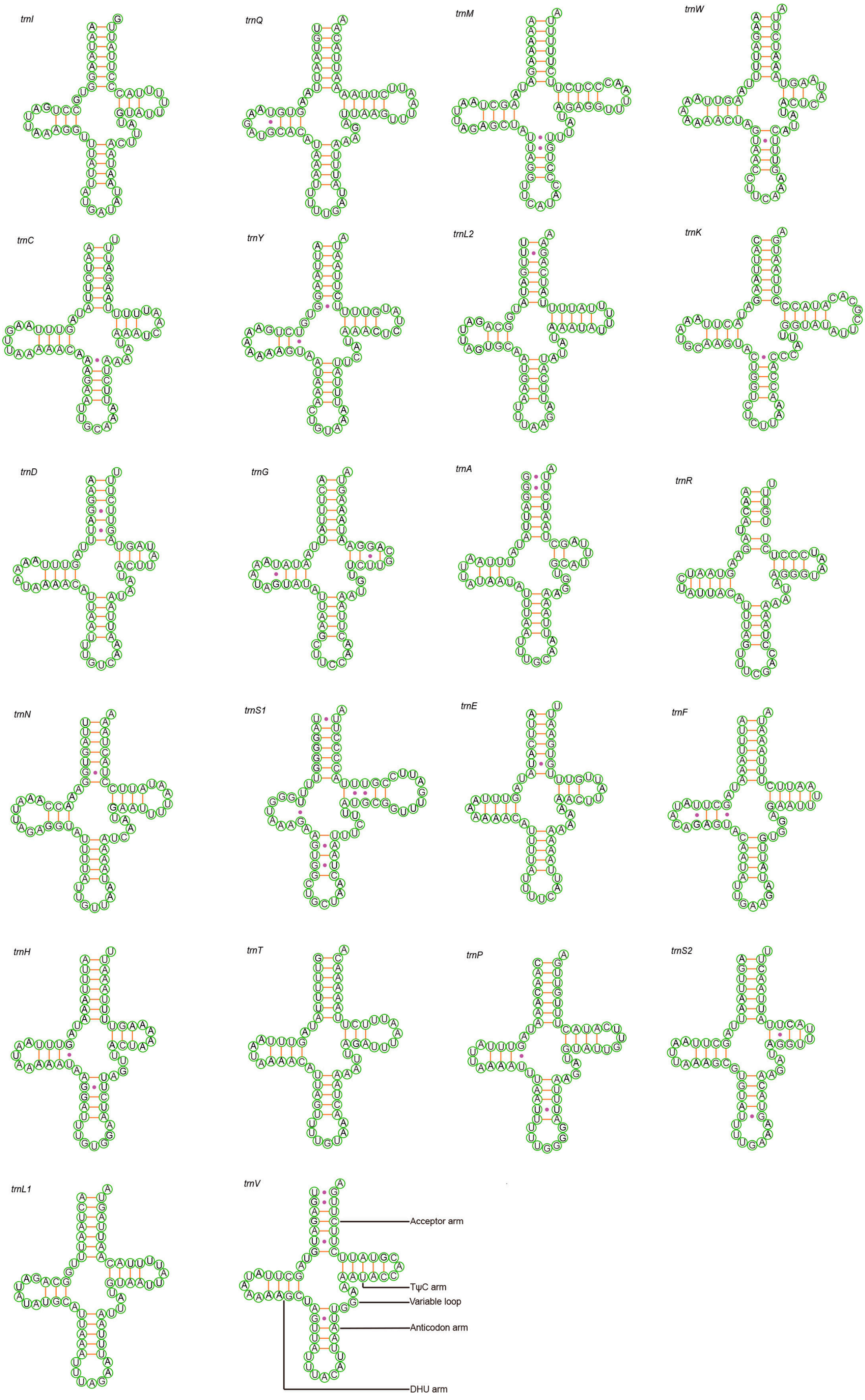

Figure S6. Predicted secondary cloverleaf structure for the tRNAs of *Empoasca serrata*.

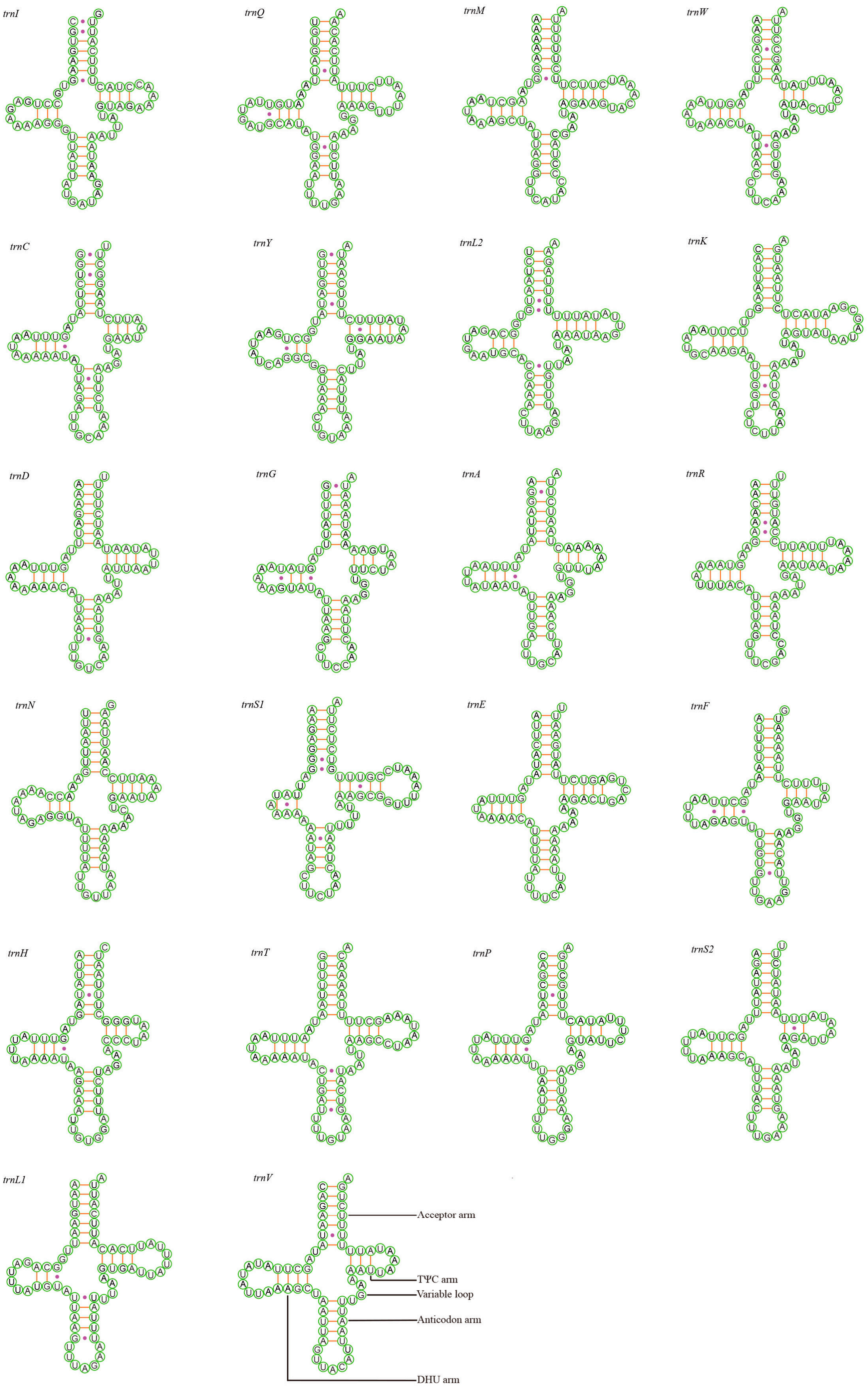

Figure S7. Predicted secondary cloverleaf structure for the tRNAs of *Eupteryx* (*E.*) *adspersa*.

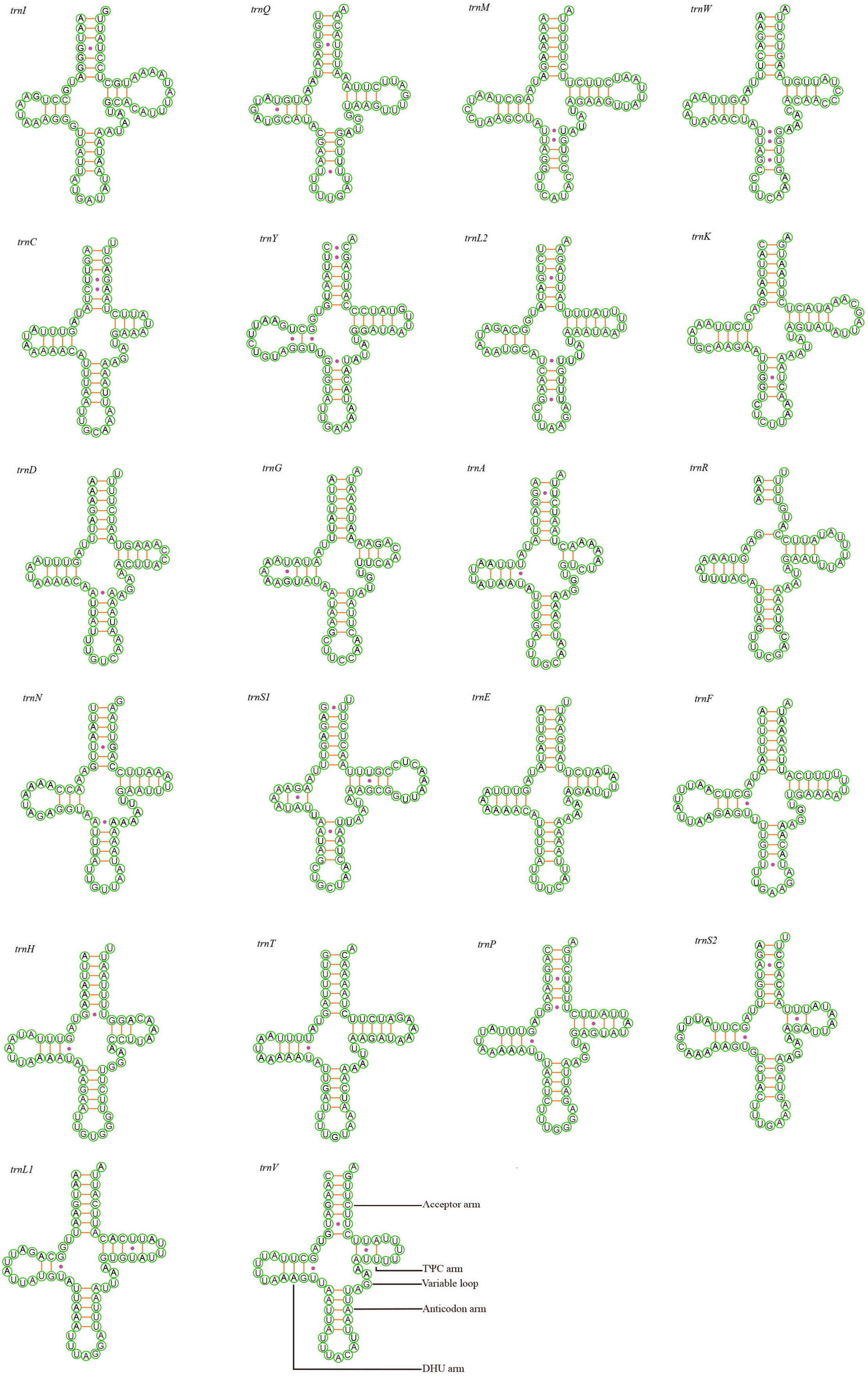

Figure S8. Predicted secondary cloverleaf structure for the tRNAs of *Eurhadina jarrary*.

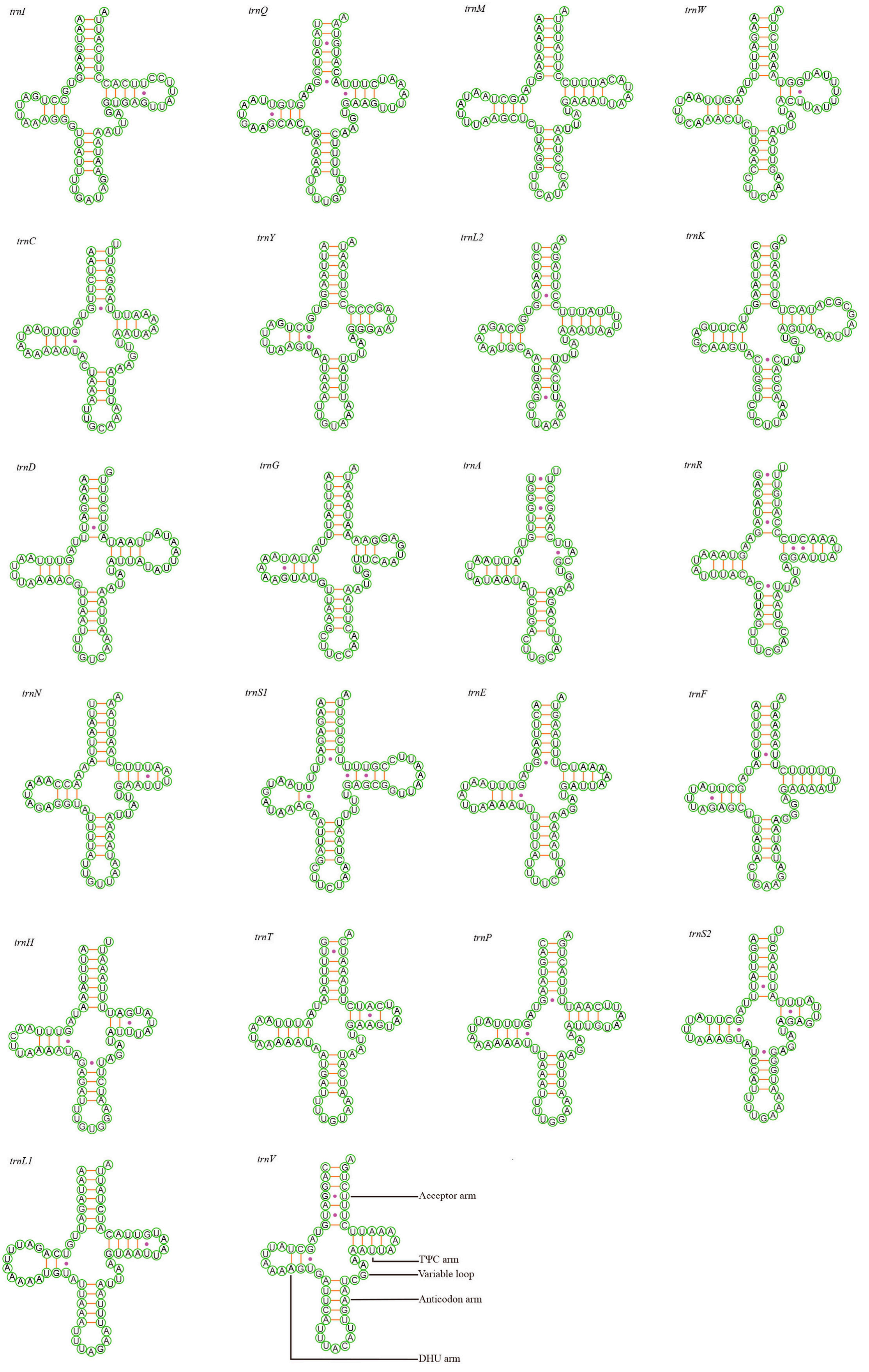

Figure S9. Predicted secondary cloverleaf structure for the tRNAs of *Kaukania anser*.

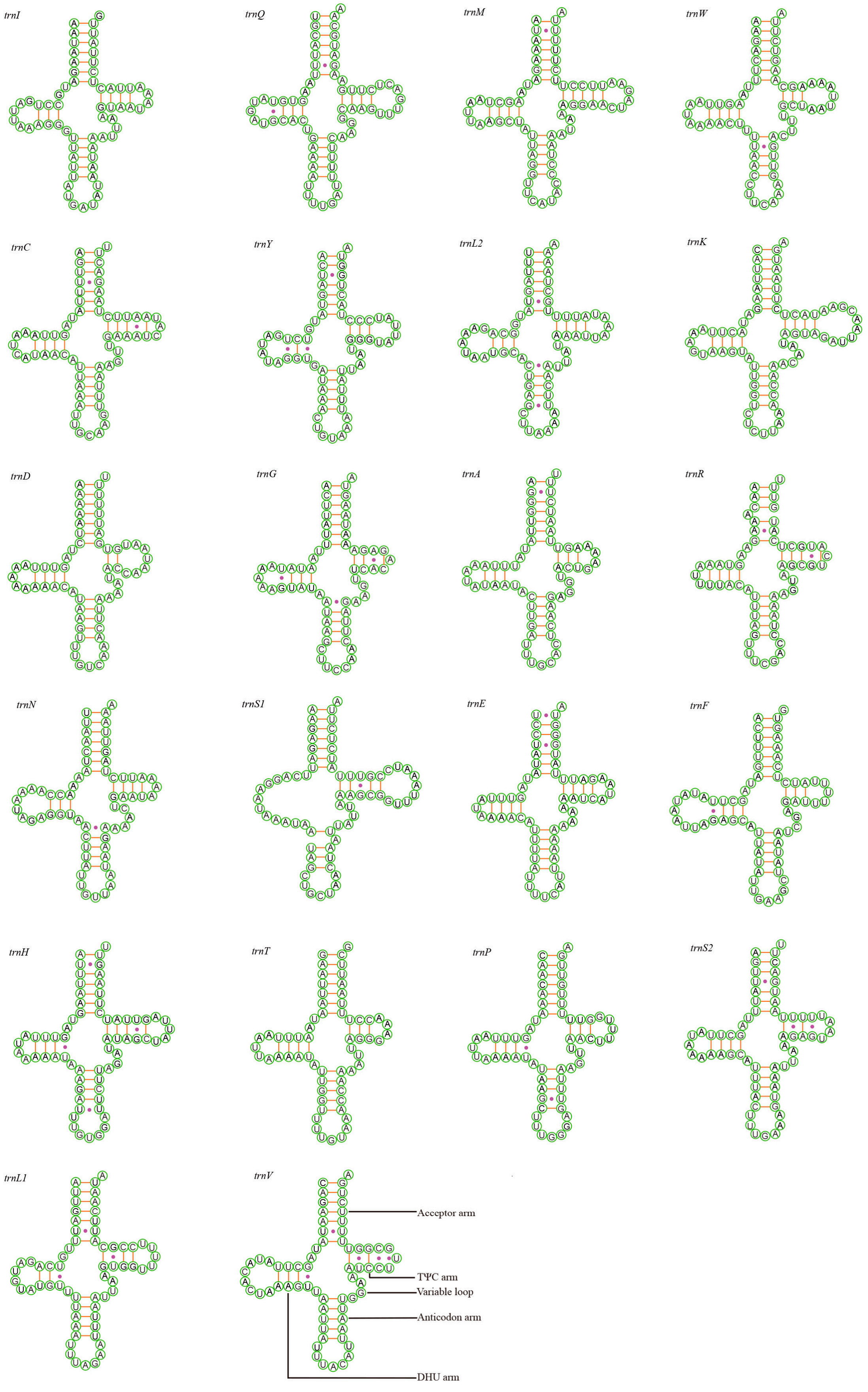

Figure S10. Predicted secondary cloverleaf structure for the tRNAs of Dikraneurini sp..

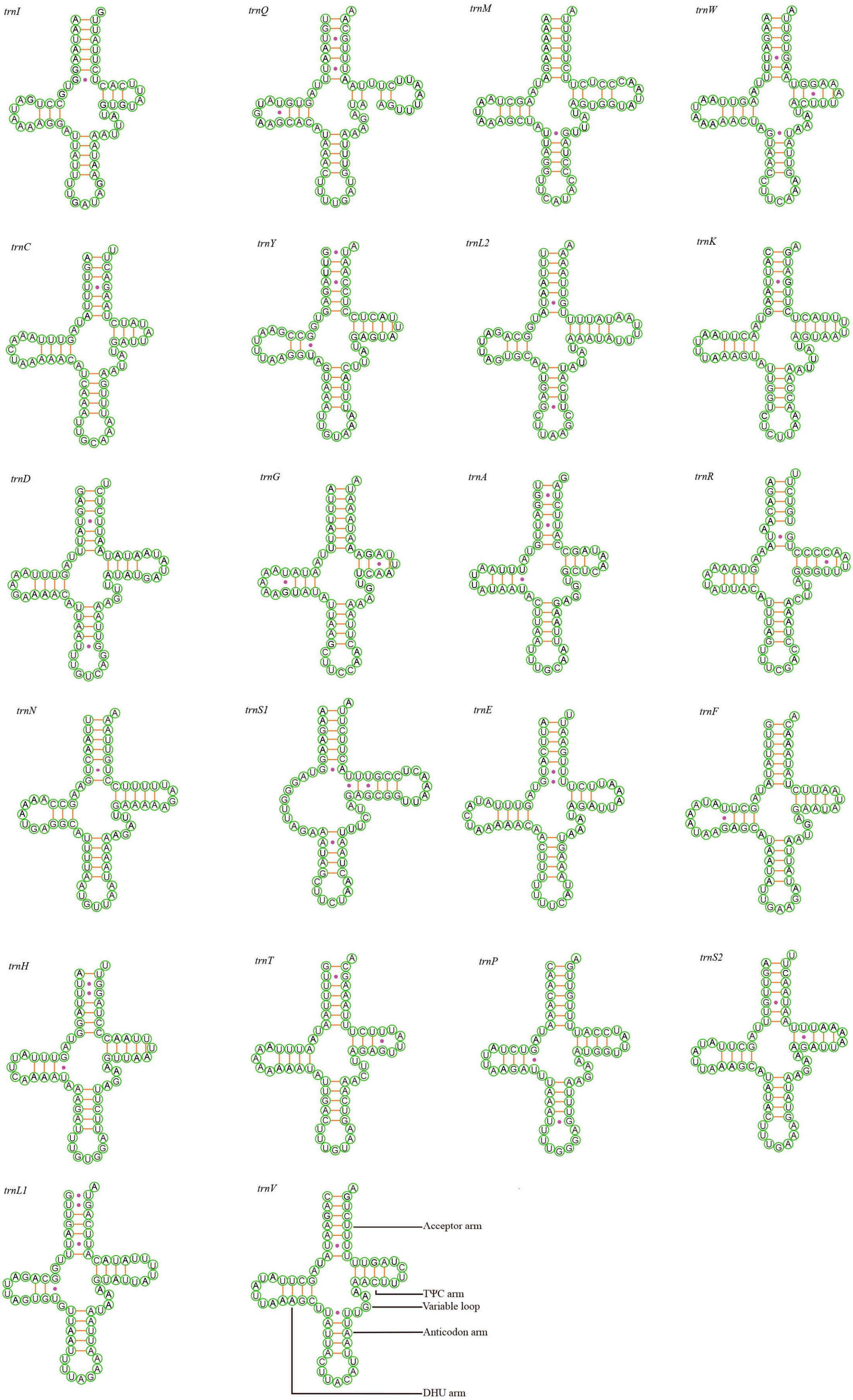

Figure S11. Predicted secondary cloverleaf structure for the tRNAs of *Shaddai* sp..

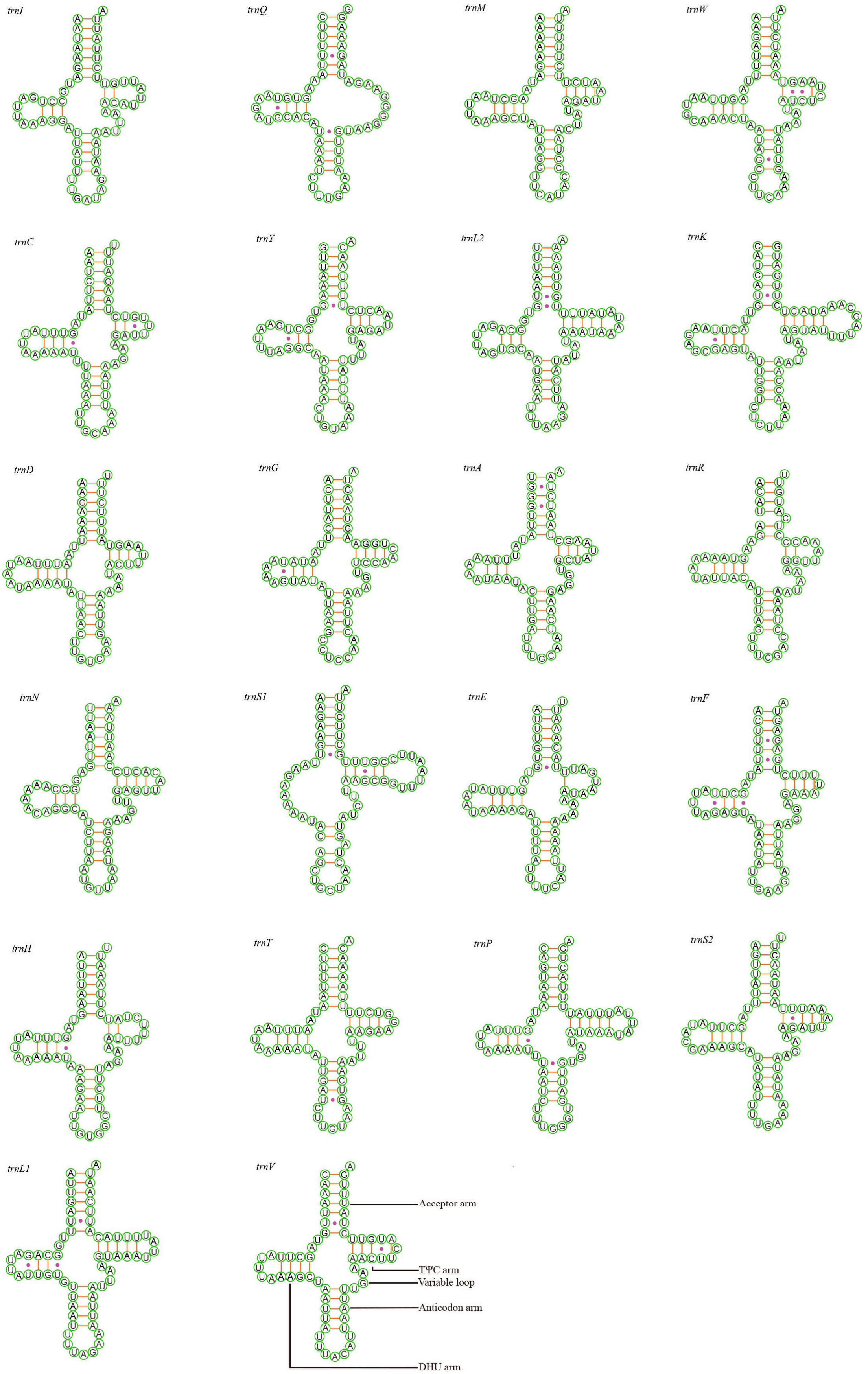

Figure S12. Predicted secondary cloverleaf structure for the tRNAs of *Sobrala* sp..

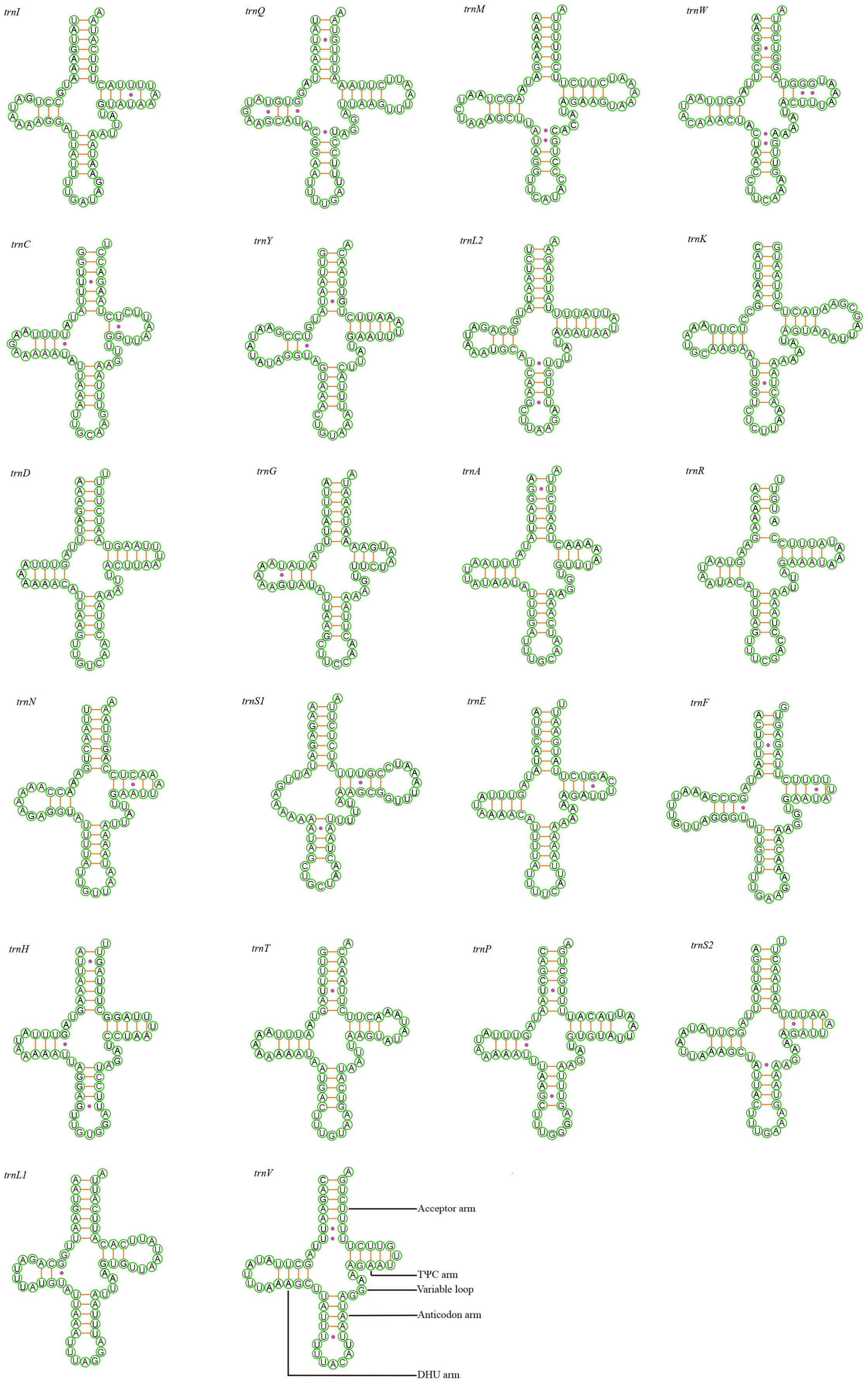

Figure S13. Predicted secondary cloverleaf structure for the tRNAs of *Yangisunda tiani*.

Table S6. Overlapping Sequences and Intergenic Spacers. Note: (*Shaddai* sp. (*S1.*); *Sobrala* sp. (*S2.*); *Dikraneura* (*D.*) *zlata* (*D1.*); Dikraneurini sp. (*D2.*); *Alebroides salicis* (*A.*); *Empoasca serrata* (*E1.*); *Elbelus tripunctatus* (*E2.*); *Kaukania anser* (*K.*); *Eupteryx* (*E.*) *adspersa* (*E3.*); *Eurhadina jarrary* (*E4.*); *Yangisunda tiani* (*Y.*)).

Genes	Gene overlaps and intergenic spacers										
	S1.	*S2.*	*D1.*	*D2.*	*A.*	*E1.*	*E2.*	*K.*	*E3.*	*E4.*	*Y.*
trnI	0	0	0	0	0	0	0	0	0	0	0
trnQ	-3	22	-3	-3	-3	-3	-3	1	-3	-3	0
trnM	1	-6	-1	0	1	-1	4	-1	-1	-1	-1
nad2	36	0	0	0	0	0	39	0	0	0	0
trnW	-2	-2	-2	-2	-2	-2	2	-1	7	14	3
trnC	-8	-8	-8	-8	-8	-8	-8	-8	-8	-8	-8
trnY	0	2	0	5	0	3	1	3	2	10	3
cox1	11	3	6	16	0	1	3	7	1	1	2
trnL2	8	-5	0	-5	0	0	4	0	0	0	0
cox2	6	0	0	0	0	0	18	0	0	0	0
trnK	0	0	0	0	0	0	0	0	0	0	0
trnD	0	0	1	1	0	0	12	6	0	2	0
atp8	-1	-2	-1	0	0	0	-2	-8	0	-1	-1
atp6	-4	-7	-7	-7	-7	-7	-4	-7	-7	-7	-7
cox3	2	0	1	0	0	0	0	3	4	1	2
trnG	-1	0	4	4	1	-1	0	6	-3	0	0
nad3	0	0	0	0	0	0	15	0	0	0	0
trnA	0	1	-2	3	-1	2	2	2	0	-1	-1
trnR	-2	-1	2	0	2	2	8	4	5	2	0
trnN	7	0	-2	-1	-1	-1	0	-1	-1	-2	0
trnS1	-1	-1	-1	-1	-1	-1	-1	-1	-3	-1	-1
trnE	-1	1	5	-1	-1	-1	2	1	5	3	0
trnF	-1	-1	6	8	-1	-1	-1	2	0	0	0
nad5	2	0	0	0	0	0	-1	0	0	0	0
trnH	0	-3	-1	0	0	-1	57	0	20	14	15
nad4	-1	-1	0	0	0	-1	0	0	-1	-1	-1
nad4L	41	-7	-7	-7	-7	-7	-7	-7	-7	-7	-7
trnT	2	2	-2	6	2	2	5	2	2	2	2
trnP	0	0	0	0	0	0	0	0	0	1	0
nad6	11	2	2	2	2	2	8	2	2	15	3
cytb	-1	-1	-8	-8	-8	-8	-1	5	-8	3	4
trnS2	-2	-2	5	-1	-2	-1	0	-2	-2	-2	-2
nad1	-1	-4	-2	-10	17	-7	-7	-7	-1	-1	-1
trnL1	0	0	0	0	0	0	-3	0	0	0	6
rrnL	0	0	0	0	0	0	0	0	0	0	0
trnV	0	0	0	0	0	0	0	0	0	0	0
rrnS	0	0	0	0	0	0	0	0	0	0	0
CR	0	0	0	0	0	0	0	0	0	0	0

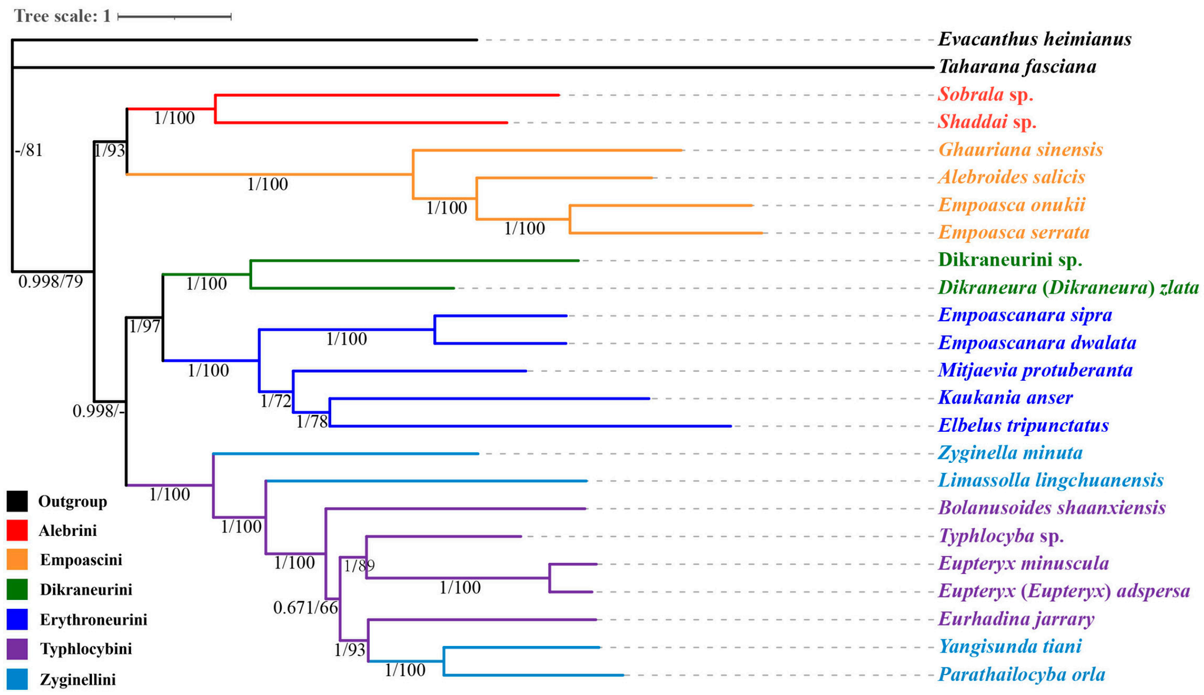

Figure S14. The phylogenetic tree produced using BI methods based on the dataset of PCG123R. ML and BI analyses showed the same topology. The numbers under the branches are Bayesian posterior probabilities (PP) and bootstrap support values (BS).

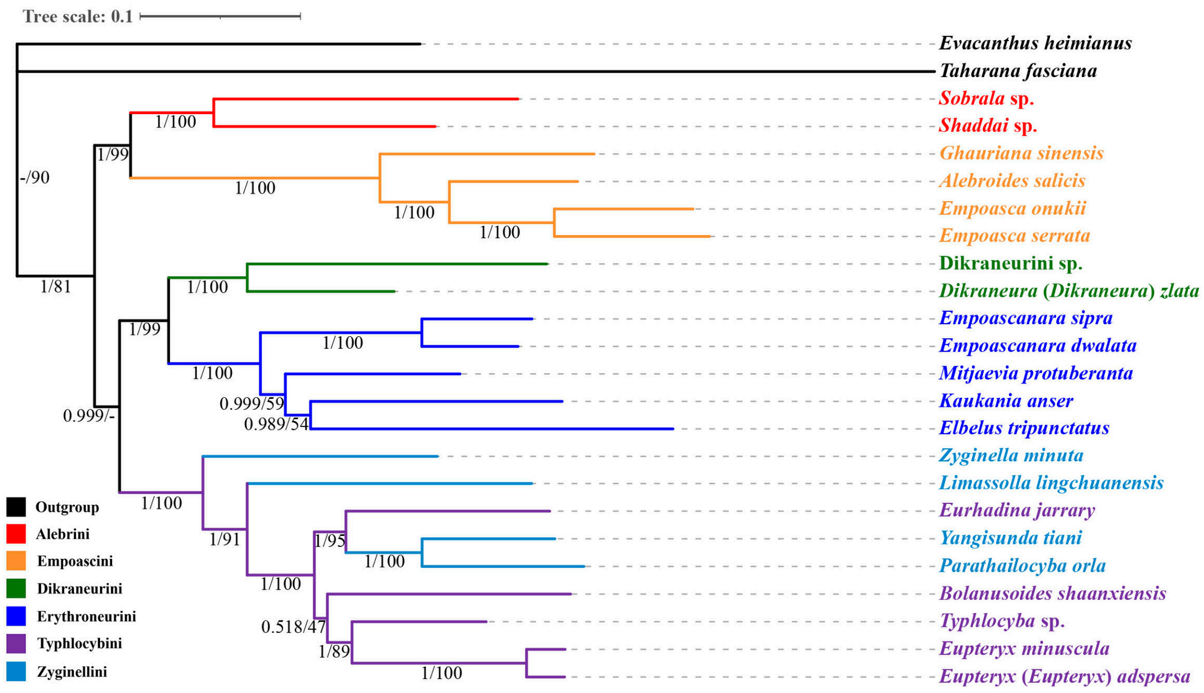

Figure S15. The phylogenetic tree produced using BI methods based on the dataset of PCG12. ML and BI analyses showed the same topology. The numbers under the branches are Bayesian posterior probabilities (PP) and bootstrap support values (BS).
